# Fas/FasL Signaling Regulates CD8 Expression During Exposure to Self-Antigens

**DOI:** 10.3389/fimmu.2021.635862

**Published:** 2021-03-24

**Authors:** Giovanna Flores-Mendoza, Noé Rodríguez-Rodríguez, Rosa M. Rubio, Iris K. Madera-Salcedo, Florencia Rosetti, José C. Crispín

**Affiliations:** ^1^ Departamento de Inmunología y Reumatología, Instituto Nacional de Ciencias Médicas y Nutrición Salvador Zubirán, Mexico City, Mexico; ^2^ Posgrado en Ciencias Biológicas, Universidad Nacional Autónoma de México (UNAM), Mexico City, Mexico; ^3^ Escuela de Medicina y Ciencias de la Salud, Tecnologico de Monterrey, Monterrey, Mexico

**Keywords:** CD8, CD95, Fas, FasL, tolerance, double negative T cell

## Abstract

Activation of self-reactive CD8^+^ T cells induces a peripheral tolerance mechanism that involves loss of CD8 expression. Because genetic deficiency of *Fas* and *Fasl* causes the accumulation of double-negative (DN; CD3^+^ TCR-αβ^+^ CD4^-^ CD8^-^) T cells that have been proposed to derive from CD8^+^ cells, we decided to explore the role of Fas and FasL in self-antigen-induced CD8 downregulation. To this end, we quantified Fas and FasL induction by different stimuli and analyzed the effects of Fas/FasL deficiency during a protective immune response and after exposure to self-antigens. Our data describes how Fas and FasL upregulation differs depending on the setting of CD8 T cell activation and demonstrates that Fas/FasL signaling maintains CD8 expression during repetitive antigen stimulation and following self-antigen encounter. Together, our results reveal an unexpected role of Fas/FasL signaling and offer a new insight into the role of these molecules in the regulation of immune tolerance.

## Introduction

Fas (CD95) and FasL play essential roles in immune function that include induction of apoptosis and modulation of T cell activation (1). In mice, *Fas* deficiency causes abnormal accumulation of antigen-specific T cells during chronic (but not acute) viral infections and after T cell activation under steady state conditions (2–4). In addition, loss of function (LOF) mutations in the genes that encode Fas and FasL cause ALPS (autoimmune lymphoproliferative syndrome), a lymphoproliferative disease associated with pathological autoimmunity (5). Therefore, Fas and FasL are thought to contribute to the control of lymphoid proliferation and the maintenance of immune tolerance.

It has been proposed that lack of Fas-mediated apoptosis represents the main mechanism behind lymphoid cell accumulation in patients with ALPS (4, 6). However, Fas also plays complex non-apoptotic roles in T cells, where, depending on the context, it can promote or inhibit activation and effector differentiation (7–10). A prominent feature of humans and mice with *Fas* or *Fasl* LOF mutations is the accumulation of an unusual population of CD3^+^ TCR-αβ^+^ T cells that lack CD4 and CD8 (double negative; DN) (11). Because their accumulation is associated with Fas deficiency, DN T cells are thought to represent products of failed T cell apoptosis (1, 4, 12, 13). However, two lines of evidence argue against this being the only mechanism for DN T cell accumulation: (a) in non-autoimmune mice, T cell-specific deficiency of *Fas* did not cause the accumulation of DN T cells (14); (b) a point mutation that avoided Fas palmitoylation, and therefore its recruitment into lipid rafts, abolished Fas-mediated apoptosis, but did not cause an increase in DN T cells (9). Consequently, the capacity of T cells to undergo Fas-mediated apoptosis and the accumulation of DN T cells do not seem to be mechanistically connected. This aspect of DN T cell biology holds particular relevance considering their possible role in autoimmunity (15, 16), allograft rejection (17), and anti-tumor immunity (18).

A wealth of evidence indicates that DN T cells derive from CD8αβ^+^ T cells: (a) CD8^+^ and DN T cells share Vβ usage and CDR3 sequences (19); (b) mice deficient in β2-microglobulin or MHC-I molecules have reduced numbers of DN T cells (20–22); (c) the *Cd8a* locus is hypomethylated in DN T cells, indicating previous transcriptional activity (23, 24); (d) CD8^+^ T cells lose CD8 when they encounter cognate antigen presented as self (25, 26); (e) DN T cells can upregulate CD8 when they undergo homeostatic proliferation under lymphopenia (27). Importantly, generation of DN T cells is not limited to situations in which Fas/FasL function is compromised, as an increased abundance of DN T cells has been reported in a number of chronic inflammatory conditions that include systemic lupus erythematosus (15), primary Sjögren’s syndrome (28), and psoriasis (29). Therefore, regulation of CD8 expression may represent an underestimated mechanism of controlling CD8 T cell function (30–32), particularly in the setting of self-antigen encounter and chronic inflammation, and the accumulation of DN T cells in patients or animals that lack Fas or FasL suggests that signaling through these molecules regulates CD8 expression. In this work, we addressed this question, using a genetic approach, to determine the role of Fas/FasL in the regulation of CD8 expression during protective and tolerance-inducing immune responses.

## Materials and Methods

### Mice

B6.MRL-*Fas^lpr^*/J (B6.*lpr*), B6Smn.C3-*Fasl^gld^*/J (B6.*gld*), C57BL/6-Tg(*TcraTcrb*)1100Mjb/J (OT-I), and C57BL/6-Tg(CAG-OVAL)916Jen/J (Act-mOVA) mice were purchased from The Jackson Laboratory (Bar Harbor, Maine, USA). B6.*lpr* OT-I, B6.*gld* OT-I, and B6.*lpr*/*gld* OT-I were generated by breeding. Mice were housed in SPF conditions on a 12 hour light/dark cycle and had *ad libitum* access to food and water. All experiments involving mice were approved by the Animal Care and Use Committee of the Instituto Nacional de Ciencias Médicas y Nutrición Salvador Zubirán (IRE-1725).

### *In Vitro* T Cell Activation

Bone marrow-derived dendritic cells (BM-DCs) were differentiated from WT, B6.*lpr*, or B6.*gld* bone marrow cell suspensions by culturing them in full RPMI (10% FBS) in the presence of GM-CSF (20 ng/mL; Peprotech) during 8 days. BM-DCs (5 x 10^4^), loaded with the indicated concentration of SIINFEKL, were cultured in U-bottom 96-well plates with 2 x 10^5^ CD8^+^ T cells isolated using CD8α^+^ T Cell Isolation Kit II (Miltenyi Biotec) from OT-I mice in B6 (WT), B6.*lpr*, or B6.*gld* background. For activation with antibodies, 1.25 x 10^6^ OT-I cells were cultured in 48-well plates coated with anti-CD3 and anti-CD28 (2 µg/mL). For quantification of gene expression by RT-qPCR, live T cells were sorted and lysed in TRIzol. Total RNA was reverse transcribed using the High-Capacity cDNA Reverse Transcription Kit (Applied Biosystems) and qPCR was performed using SYBR Green PCR Master Mix (Applied Biosystems). Results were normalized using *Actb* and are expressed as ΔCt.

### Adoptive Transfer

OT-I cells were adoptively transferred by i.v. injection. One-day after, 10^4^ c.f.u. of *Listeria monocytogenes* expressing recombinant OVA (LM-OVA; a generous gift from Dr. Michael J. Bevan, University of Washington) were injected i.v (33). For exposure to self-antigens, OT-I cells were adoptively transferred (i.v.) into Act-mOVA or B6 (control) mice. Transferred cells were analyzed in spleens of recipient mice at the indicated days after injection.

### Flow Cytometry

Labeled antibodies were purchased from Tonbo, Biolegend, and eBiosciences. (**Supplemental Table 1**). For staining, 1 x 10^6^ cells were incubated with antibodies (1:100), at room temperature, for 30 min. Cells were washed twice and resuspended in PBS + 2% FCS and acquired in a FACS Aria II instrument (BD Biosciences). Data was analyzed using FlowJo software.

### Statistics

Statistical tests were calculated using Microsoft Excel and GraphPad Prism. The statistical test used and P values are indicated in each figure. In general, for comparison between two groups, paired or non-paired Student’s t test was used. To compare more than 2 groups, one-way ANOVA was used. P values <0.05 were considered significant.

## Results

### The Kinetics of Fas and FasL Expression Vary According to the Activation Stimulus

**Figure 1:**
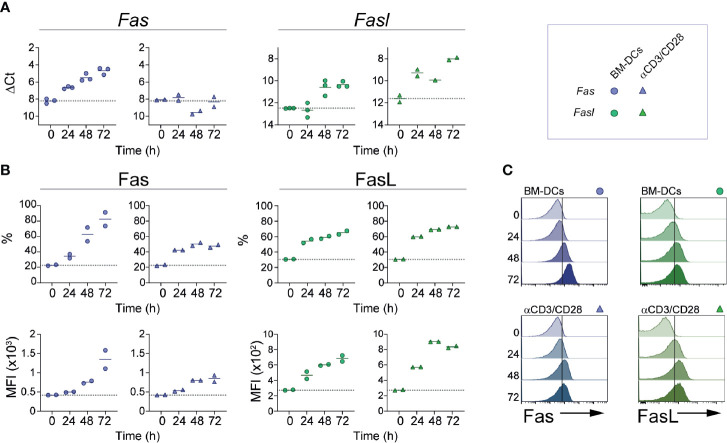
Fas and FasL expression kinetics. CD8^+^ OT-I cells were activated *in vitro* using bone marrow-derived dendritic cells (BM-DCs; circles) loaded with SIINFEKL (1 mM) or with plate-bound anti-CD3 and anti-CD28 (triangles). At the indicated time points, cells were lysed for RNA extraction **(A)** or stained for flow cytometry **(B, C)**. **(A)** *Fas* and *Fasl* relative expression (ΔCt). **(B)** Fas and FasL expression is shown as fraction of positive cells (%) and mean fluorescence intensity (MFI) of positive cells. **(C)** Representative histograms of **(B)**. Each symbol **(A, B)** represents one experiment done with pooled cells from 2-3 mice. Solid lines indicate mean; dotted line indicate the expression level of unstimulated cells (0).

Expression of Fas and FasL on naïve CD8 T cells is minimal, but levels of both molecules increase following cell activation (34, 35). To determine the kinetics with which these receptors are induced by activation on CD8^+^ T cells, we isolated OT-I cells and activated them *in vitro* using plate-bound anti-CD3 and anti-CD28, or in the presence of BM-DCs loaded with the ovalbumin-derived peptide SIINFEKL. As shown in **Figure 1A**, activation of CD8 T cells with BM-DCs induced robust transcription of *Fas* that initiated 24 h after cell activation and reached an ~8 fold increase at 72 h. In contrast, cell activation with anti-CD3 and anti-CD28 did not induce a detectable increase in *Fas* expression at the mRNA level. Transcription of *Fasl* was promoted by both types of cell activation, but appeared earlier and reached higher levels in cells stimulated by anti-CD3 and anti-CD28. When analyzed at the protein level, expression of surface Fas and FasL reflected the regulation at the mRNA level (**Figures 1B, C**). Fas was induced more strongly by BM-DCs and its levels increased gradually during the observed period of time, whereas FasL was similarly induced by BM-DCs and the combination of anti-CD3 and anti-CD28. These results indicate that in CD8^+^ T cells, Fas and FasL expression is regulated, at least partially, at the transcriptional level, and is affected by signals present during cell activation.

### Fas-FasL Signaling Maintains CD8 Expression Levels During Cell Re-Stimulation

**Figure 2:**
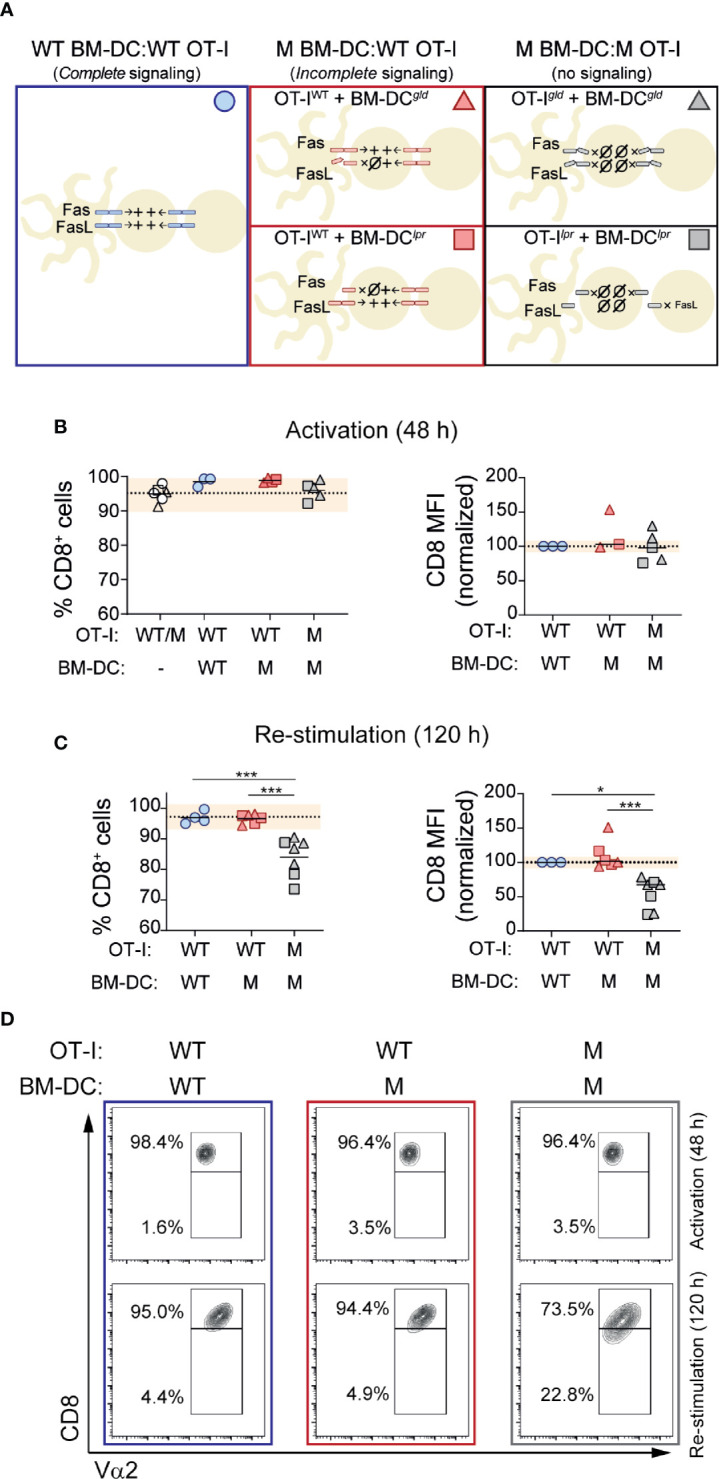
Fas-FasL signaling maintains CD8 expression during re-stimulation. CD8^+^ OT-I cells were activated in the presence of BM-DCs loaded with SIINFEKL (1 mM). After 48 hours (activation), CD8 expression levels were quantified by flow cytometry in an aliquot of the cells. The rest were placed in a new plate with fresh BM-DCs (in the presence of SIINFEKL). CD8 expression was reevaluated after 72 hours (120 h re-stimulation). **(A)** Experimental strategy indicating the presence of Fas-FasL signaling in the different cell culture conditions. **(B, C)** CD8 expression after the initial activation (48 h) and after re-stimulation (120 h). Wild-type (WT) or mutant (M; B6.*lpr* or B6.*gld*) OT-I cells were activated in the presence of WT or M BM-DCs, as indicated. Percentage of CD8^+^ cells (left) or CD8 expression per cell (mean fluorescence intensity; MFI; right) on OT-I cells is shown. Each symbol represents one experiment done with pooled cells from 2-3 mice. Circles represent the conditions where OT-I cells and BM-DCs were WT; triangles when the mutant cells were B6.*gld*; squares when the mutant cells were B6.*lpr*. Dotted lines indicate mean of WT cells; shaded area represents range of WT cells. **P < 0.01; ***P < 0.001 (one-way ANOVA). **(D)** Representative contour plots of OT-I cells after 48 and 120 hours of activation and re-stimulation, respectively.

Genetic deficiency of *Fas* or *Fasl* causes massive accumulation of TCR-αβ DN T cells, suggesting that signaling through these molecules may contribute to CD8 expression (9, 10, 36, 37). Previous work from our group has shown that CD8 expression is lost in CD8 T cells transferred into mice that locally or ubiquitously express the CD8^+^ T cell cognate antigen as self (25, 26). In that context, several aspects could contribute to CD8 loss. These include factors related to antigen presentation (32, 38) and to the repetitive nature of the antigen encounter (39, 40). To explore this process, and in particular to analyze whether signaling through Fas and/or FasL plays a role in maintaining CD8 expression, we designed an *in vitro* system that would allow us to evaluate the role that repetitive cognate antigen stimulation and signaling through Fas and FasL may exert on CD8 expression during T cell activation. To this end, we took advantage of B6.*lpr* and B6.*gld* mice. B6.*lpr* mice are homozygous for a mutation in *Fas*, caused by the insertion of the ETn retrotransposon that abolishes the expression of the gene (41). B6.*gld* mice have a point mutation near the C-terminal region of FasL that affects its ability to bind Fas (42). Thus, B6.*lpr* cells lack Fas and B6.*gld* cells express a FasL variant that cannot engage Fas. This allowed us to compare the activation of OT-I cells in the presence of WT BM-DCs or BM-DCs lacking Fas (B6.*lpr*) or functional FasL (B6.*gld*). By crossing OT-I mice with B6.*lpr* and B6.*gld* mice, we obtained OT-I cells deficient in Fas and FasL. These cells allowed us to analyze CD8^+^ T cell activation in cell culture systems devoid of signaling through Fas and FasL (**Figure 2A**). To observe the effects of repetitive antigen encounter, we setup a two-step stimulation system, where we incubated OT-I cells in the presence of cognate antigen-loaded BM-DCs during 48 h and then we replated the OT-I cells with fresh BM-DCs. We analyzed CD8 expression on OT-I cells after the initial stimulation (activation) and after 72 h of re-stimulation.

We considered CD8 expression as percentage of CD8 positive cells within OT-I cells (% CD8^+^) and as CD8 abundance per cell (CD8 mean fluorescence intensity; MFI). As shown in **Figure 2B**, cells maintained CD8 expression during the initial 48 h activation period, and lack of BM-DC→T cell signaling (through Fas or FasL) did not affect CD8 expression. Likewise, activation of B6.*gld* OT-I cells in the presence of B6.*gld* BM-DCs, or B6.*lpr* OT-I cells with B6.*lpr* BM-DCs (no Fas or FasL signaling; **Figure 2A**) had no effect on CD8 levels during the initial 48 h stimulation period (**Figure 2B**).

Re-exposure to cognate antigen had no effects on CD8 expression on WT OT-I cells, activated and re-stimulated by WT or by mutant (M) BM-DCs, indicating that BM-DC→T cell signaling through Fas or FasL does not play an essential role in the modulation of CD8 expression during T cell activation or re-stimulation (**Figure 2C**). In contrast, complete lack of Fas/FasL signaling, was associated with a modest, albeit consistent and statistically significant decrease in CD8 expression, quantified as the fraction of CD8^+^ cells or as CD8 levels per cell (**Figures 2C, D**).

Because Fas and FasL have been shown to modulate TCR signaling (7, 8), we analyzed the role of cognate peptide concentration during OT-I activation and re-stimulation. CD8 T cell stimulation with higher concentrations of SIINFEKL tended to decrease the expression of CD8 and of the TCR, but only after re-stimulation. This effect was more marked in the absence of Fas and FasL signaling (**Supplemental Figure 1**). This suggests that signaling through Fas and/or FasL signaling may decrease the strength of TCR signaling during repetitive encounters with cognate antigen.

### Loss of Fas/FasL *cis* Signaling Does Not Affect CD8 Loss *In Vivo*

**Figure 3:**
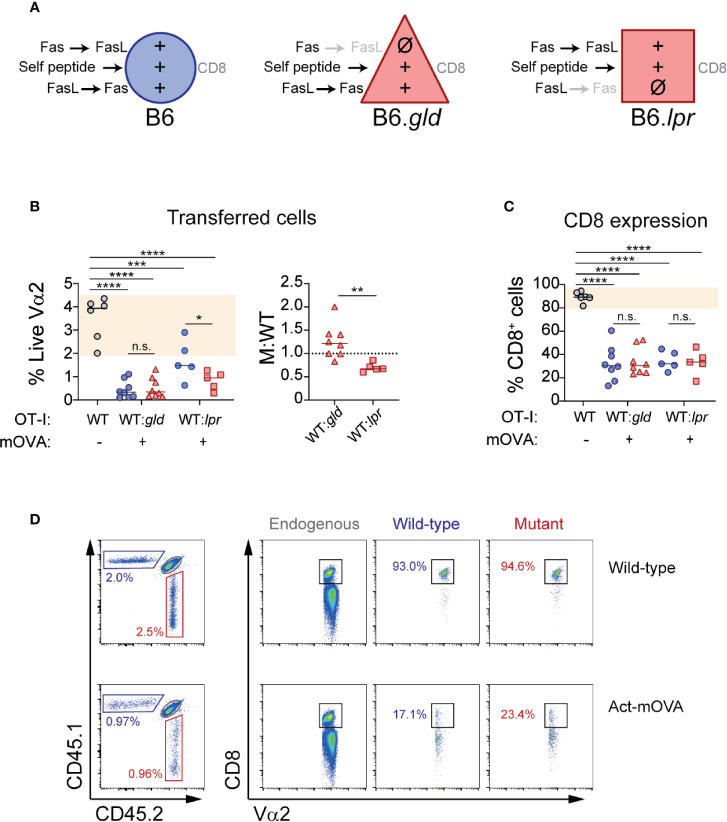
T cell-specific deficiency of Fas or FasL does not affect CD8 loss during self-antigen exposure. Wild-type CD45.1 (WT) or mutant CD45.2 (B6.*gld* or B6.*lpr*) OT-I cells were adoptively transferred (1:1 ratio) into CD45.1/2 mice that ubiquitously express ovalbumin (Act-mOVA), or into control CD45.1/2 mice. Seven days later, transferred cells and CD8 expression were analyzed. **(A)** Experimental layout, indicating the symbols that represent each cell type (in **B**, **C**) and the signals received by each cell during exposure to self-antigens. **(B)** Abundance of transferred cells, shown as the percentage of CD45.1 (WT) or CD45.2 (B6.*gld* or B6.*lpr*) cells within live Vα2^+^ T cells (left), or as M:WT ratio (right). **(C)** Fraction of transferred cells positive for CD8. Each symbol **(B, C)** represents one mouse. Solid lines indicate mean. Shaded area indicates range of WT cells. **(C)** Representative dot plots showing transferred cells (left) and CD8 expression (right) in a WT and an Act-mOVA mouse. ***P<0.001; ****P<0.0001 (one-way ANOVA). **P<0.01 (unpaired t test).

The results from our *in vitro* experiments showed unaltered CD8 expression in the presence of one-way BM-DC→T cells Fas or FasL signaling. To determine whether the absence of Fas or FasL on CD8 T cells affected the behavior of OT-I cells adoptively transferred into mice that ubiquitously express their cognate antigen, we co-transferred WT OT-I (CD45.1) and B6.*gld* or B6.*lpr* OT-I (CD45.2) into CD45.1/2 Act-mOVA mice in a 1:1 ratio (**Figure 3A**). As expected, the presence of OVA was associated with a contraction of transferred antigen-specific cells. However, absence of Fas and FasL affected CD8 T cell contraction in a different manner. Whereas lack of FasL did not affect self-antigen-induced contraction and actually tended to increase the number of OT-I cells at day 7 post-transfer, lack of Fas was associated with a significant decrease in the number of live OT-I cells suggesting that signaling through Fas may in fact promote cell survival in CD8 T cells exposed to self-antigen (**Figure 3B**). When we analyzed CD8 downregulation, our results in this *in vivo* setting were analogous to the ones obtained in our *in vitro* re-stimulation system. B6.*gld* as well as B6.*lpr* OT-I cells downregulated CD8 in a normal manner, not different than the WT OT-I cells they were cotransferred with (**Figures 3C, D**).

These experiments demonstrate that Fas and FasL expression on T cells is dispensable for self-antigen-induced contraction of CD8 T cells. In fact, lack of T cell expression of Fas caused an unexpected drop in the numbers of live OT-I cells transferred into Act-mOVA mice, suggesting that signaling through Fas may directly or indirectly promote T cell survival in this setting. In concordance with the results of our *in vitro* experiments, when Fas or FasL deficiency was limited to T cells, CD8 downregulation induced by self-antigen was not affected.

### Fas/FasL Signaling Maintains Cell Numbers and CD8 Expression During Encounter With Self-Antigens

**Figure 4:**
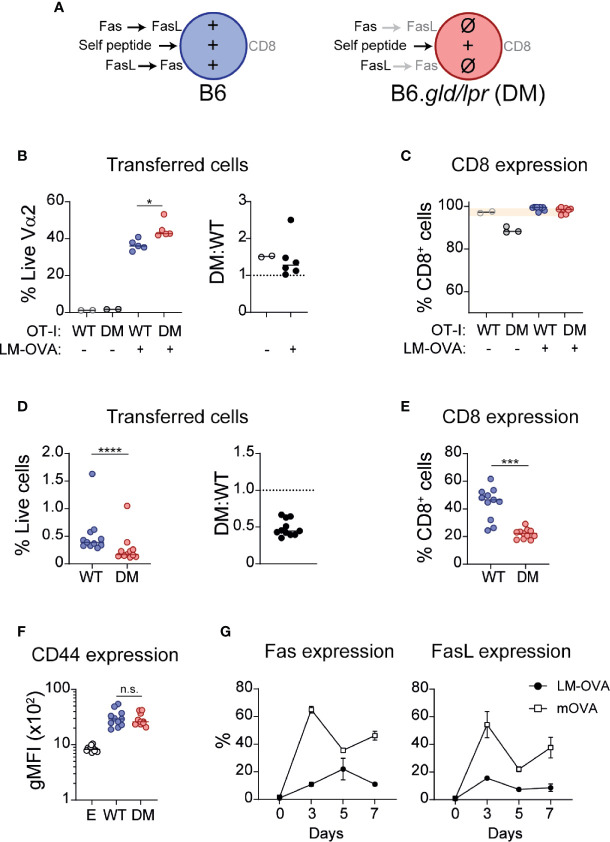
Fas-FasL signaling maintains CD8 expression during self-antigen encounter. **(A)** Experimental layout, indicating the symbols that represent each cell type (in **B–F**) and the signals received by each cell during exposure to self-antigens. **(B, C)** WT (CD45.1/2) and double mutant (DM; B6.*lpr*/*gld*; CD45.2) OT-I cells were adoptively transferred (1:1) into CD45.1 mice. The next day, mice were infected with ovalbumin-expressing Listeria monocytogenes (LM-OVA). At Day 7 post-infection, mice were sacrificed and cell abundance and CD8 expression were analyzed. **(B)** Abundance of transferred cells, shown as the percentage of WT or DM cells within live Vα2^+^ T cells (left), or as DM : WT ratio (right). **(C)** CD8 expression on transferred cells. **(D–F)** WT and DM cells were adoptively transferred (1:1) into mice that ubiquitously express OVA (Act-mOVA) and analyzed 7 days later. **(D)** Abundance of transferred cells. **(E)** CD8 expression on WT and DM cells. **(F)** CD44 expression (gMFI) in endogenous **(E)** and transferred cells. Each symbol represents one mouse. Solid lines indicate mean. * *P*<0.05 (one-way ANOVA). ***P ≤ 0.001; ****P<0.0001 (paired t test). **(G)** Fas (left) and FasL (right) expression kinetics (mean ± SEM) in WT OT-I cells during LM-OVA infection (black circles) or self-antigen exposure (empty squares). n.s., not significant.

Collectively, the presented *in vitro* and *in vivo* data indicated that complete absence of Fas/FasL signaling was associated with loss of CD8 expression during repetitive antigen encounter, but that partial interruption of this signaling pathway had modest or no obvious effects. To confirm these observations, we generated double mutant (DM; B6.*lpr*/*gld*) OT-I mice and co-transferred WT and DM OT-I cells (1:1 ratio; **Figure 4A**) into WT recipient mice and infected them with OVA-expressing *Listeria monocytogenes* (LM-OVA). At Day 7, we quantified the abundance of WT and DM cells and analyzed their expression of CD8. As shown in **Figure 4B**, DM cells were modestly more abundant (*P*=0.016) at the peak of clonal expansion (DM : WT ratio 1.45 ± 0.22), consistent with the role of Fas/FasL in re-stimulation-induced cell death (4). However, CD8 expression remained high in WT and DM cells (**Figure 4C**). Thus, in the course of a protective immune response, during pathogen-driven T cell activation, Fas/FasL signaling is not necessary for maintaining CD8 expression, but modestly curbs clonal expansion. To analyze the role of Fas/FasL signaling during encounter with self-antigens, a situation that promotes CD8 downregulation (25, 26), we co-transferred WT and DM OT-I cells into Act-mOVA mice. In this context, lack of Fas and FasL was associated with a marked drop in cell numbers and at Day 7 post-transfer the ratio of DM : WT cells was 0.48 ± 0.03 (**Figure 4D**). Importantly, in concordance with our *in vitro* experiments, the fraction of cells that maintained CD8 expression was significantly lower within DM OT-I cells (WT 44.0% ± 3.5 vs. DM 21.89% ± 1.1, *P*=0.001) (**Figure 4E**). This was probably not associated with increased activation, as CD44 upregulation was similar in WT and DM cells (**Figure 4F**).

Because of the contrasting effects of Fas/FasL signaling during productive and tolerance-inducing immune responses, we analyzed the kinetics of Fas and FasL expression in WT OT-I cells in both scenarios. As shown in **Figure 4G**, expression of Fas increased gradually and peaked at Day 5 in OT-I cells activated in the context of LM-OVA infection. In contrast, Fas expression reached an earlier and higher peak in cells exposed to self-antigens. Analogously, induction of FasL was higher in cells exposed to self-antigens.

## Discussion

Fas and FasL play complex roles in the immune system. Present in a large variety of cells, the regulation of their expression and the consequences of their engagement vary greatly depending on the cell context in which they appear. Here, we have analyzed the effects of complete absence of Fas/FasL signaling in two different settings, during an infection with an intracellular bacterial strain and during the encounter of ubiquitous antigen presented as self. Because in both systems we used cells with the same antigenic specificity, our experimental design eliminated differences in TCR affinity, a factor commonly relevant during the comparison of self- and pathogen-derived antigens (T cells usually have lower affinity toward self-antigens than against external antigens), and allowed us to observe the behavior of OT-I cells during a protective and a tolerogenic immune response and to determine whether the absence of Fas/FasL signaling affects CD8 expression in those circumstances.

We observed that the context in which CD8 T cells are primed, affects the expression of both Fas and FasL. *In vitro* experiments showed that CD8^+^ T cell activation *via* BM-DCs induced higher expression of Fas (but not FasL) than activation of the same cells with plate-bound anti-CD3 and anti-CD28. Although these two systems are different and it is not possible to weigh the influence of TCR affinity, the fact that BM-DCs induced much higher expression of Fas, suggests that signals different to CD28 and CD3 (e.g. DC-derived cytokines and/or surface molecules) may promote transcription of Fas (43). This hypothesis is supported by previous findings that showed that T cell activation with concanavalin A induced higher levels of Fas than activation through CD3 (34). Our *in vivo* experiments further confirmed this and showed that exposure to self-antigen elicits a much stronger and earlier expression of both Fas and FasL than encounter to the same antigen in the context of bacterial infection.

In previous work we have shown that exposure of CD8^+^ T cells to self-antigen induces an inactivation program that includes the downregulation of CD8 expression (25–27). Because we never observed CD8 downregulation during *in vitro* activation and CD8 loss was only observed in tissues locally expressing the cognate antigen (25), we hypothesized that repetitive encounter with antigen –as occurs *in vivo* during self-antigen encounter- may play a role in this process and that signaling through Fas or FasL may regulate CD8 loss. We found that in WT CD8^+^ T cells, repetitive *in vitro* activation did not affect CD8 levels and, importantly, that absence of Fas or FasL on the APC had no effects. Our interpretation to this observation was that *cis* signaling (Fas and FasL on the same cell) or *trans* signaling (Fas and FasL on different T cells) could avoid CD8 downregulation because the OT-I cells did not require Fas or FasL signals originated from the APC in the presence of *cis* signaling. The importance of *cis* engagement for signaling or as competition for ligands presented in *trans*, has been observed in other T cell co-receptors, for example PD-1, PD-L1 and CD80, HVEM and BTLA, or Notch and Delta (44–49) and also in Fas-FasL during the process of memory development (36). This seems to be the case, because when Fas-deficient OT-I cells were activated in the presence of Fas-deficient BM-DCs (or FasL deficient T and DCs were used), CD8 levels decreased. These experiments suggested that Fas/FasL signaling, during repetitive antigen encounter, maintains CD8 expression. To determine the role of TCR signaling strength in this process, we activated OT-I cells with varying concentrations of SIINFEKL, in the presence or absence of Fas/FasL signaling. We observed that lack of Fas/FasL signaling increased the magnitude of CD8 and TCR downregulation induced by high antigen concentrations. These data indicate that Fas/FasL may modulate TCR signaling, particularly during repetitive encounter with high affinity antigens. Further, the fact that Fas (and not FasL) deficiency was associated with higher levels of OT-I death during adoptive transfer into Act-mOVA mice suggests that signaling through Fas may modulate TCR signaling in this context thus limiting cell death caused by exposure to persistent antigen.

Exclusive absence of Fas or FasL T cell signaling did not affect CD8 downregulation. In contrast, complete absence of Fas/FasL signaling significantly increased CD8 to DN T cell conversion. This effect could not be attributed to defective apoptosis, because as mentioned earlier, absence of Fas (either alone or combined with FasL deficiency) did not cause an accumulation of OT-I cells. Moreover, in concordance with Hao et al., that demonstrated that FasL blockade is necessary for the accumulation of DN T cells in the presence of T cell-specific *Fas* deficiency (14), we observed that whereas absence of FasL did not promote DN T cell generation when present as an isolated defect, it did robustly in the presence of the concomitant absence of Fas. This suggests that DN T cell expansion in germline mutants is a complex phenomenon where lack of Fas and FasL contribute differently. It also poses the question of whether genetic variants affecting FasL reverse signaling or the crosstalk between Fas and FasL signaling pathways could modify disease expression in patients with ALPS. This may be particularly relevant in cases where specific mutations are associated with heterogeneous phenotypes (50).

Our findings reveal an unexpected role for Fas/FasL signaling during peripheral tolerance: expression of Fas and FasL is robustly induced by self-antigen encounter and *cis* signaling through this receptor pair may protect self-reactive cells from deletion and from CD8 downregulation, perhaps by annulling Fas/FasL signaling from APCs to T cells as has been proposed in other systems (2). In contrast, absence of Fas/FasL had no effects on CD8 expression during a protective immune response induced by a bacteria. Why does absence of Fas and FasL affect so differently the fate of cells in two settings that share the CD8 T cells and the antigen? The fact that Fas and FasL induction differs greatly in these two types of antigen encounter suggests that Fas/FasL signaling may be more relevant for T cells in the context of self-antigen encounter than during responses that induce a strong clonal expansion. Numerous studies have reported that Fas and FasL exert costimulatory effects on T cells (8, 51–54). It is possible that in this system, the absence of Fas and FasL-derived costimulation impairs OT-I proliferation. It also raises the question of whether CD8^+^ and DN T cells depend differently on Fas/FasL costimulatory properties, as it has been reported that TCR signaling strength is an important modulator of FasL costimulatory and inhibitory effects (55). In patients with ALPS, CD4 and CD8 T cells exhibit abnormal phenotypes reminiscent of terminally differentiated exhausted T cells seen in conditions where T cells are chronically stimulated. This phenotype, also observed in their DN T cells, along with evidence that links the TCR repertoire in CD8^+^ and DN T cells, suggests that self-antigen encounter drives CD8 to DN T cell conversion in ALPS and emphasizes the importance of Fas in keeping in check self-reactive CD8 T cells (56, 57).

Because in our *in vivo* system we used a unique high affinity antigen, we were not able to consider the role of antigen affinity, which represents a variable that could contribute to the lymphoproliferation observed in mice that lack Fas or FasL in the presence of a diverse repertoire. Together, the evidence indicates that Fas/FasL signaling promotes CD8 expression on self-reactive T cells exposed to self-antigen, but that the degree of clonal deletion is regulated by other factors, perhaps controlled at the level of TCR signaling and therefore regulated by affinity toward the antigen. This hypothesis that would predict that the absence of Fas or FasL would favor the loss of CD8 and the accumulation of low-affinity self-reactive cells. In support of this, we observed that changes in the concentration of SIINFEKL during *in vitro* re-stimulation were inversely correlated with CD8 expression.

Because humans and mice with *Fas* or *FasL* LOF mutations lack a functional molecule in all cells, the net result is lack of signaling through Fas and through FasL. When we used double mutant (B6.*gld*/*lpr*) OT-I cells, we completely blocked all Fas and FasL signaling in T cells. However, it would be important to distinguish between the effects of Fas and FasL engagement. Theoretically, Fas-deficient OT-I cells could be adoptively transferred into FasL deficient recipient mice, to observe the effects of T cell FasL signaling in the absence of Fas signaling (or the inverse experimental setup). The main caveat of these systems is that Fas-deficient cells express FasL and thus induce apoptosis in Fas-bearing cells. Therefore, adoptive transfer of Fas-sufficient T cells into Fas-deficient animals could result in apoptosis of the transferred cells and alter the process of CD8 downregulation. On the other hand, an advantage provided by our system is the opportunity to dissect the individual contribution of Fas/FasL signaling exclusively in the context of the CD8 T cell and avoid the implications of its deficiency in other cell types.

In summary, Fas and FasL expression are differentially induced on CD8 T cells depending on the conditions that prevail during their priming. High expression of Fas and FasL induced during self-antigen presentation could regulate CD8 expression and cell survival and therefore contribute to the regulation of T cell responses to self-peptides.

## Data Availability Statement

The raw data supporting the conclusions of this article will be made available by the authors, without undue reservation.

## Ethics Statement

The animal study was reviewed and approved by the Comité Interno para el Cuidado y Uso de Animales de Laboratorio (CICUAL), Instituto Nacional de Ciencias Médicas y Nutrición Salvador Zubirán.

## Author Contributions

Conceptualization, GF-M, NR-R, FR, and JCC. Methodology, GF-M, NR-R, RMR, IM-S, FR, and JCC. Investigation, GF-M, NR-R, RMR, IM-S, and FR. Writing GF-M and JCC. Funding Acquisition JCC. Supervision, FR and JCC. All authors contributed to the article and approved the submitted version.

## Funding

This work was supported by grants from CONACYT (Consejo Nacional de Ciencia y Tecnología, Mexico) to JCC (CB-2015-256752). GF-M was supported by a doctoral fellowship (CONACYT) and this work will allow her to contend for a doctoral degree (Doctor in Science, Experimental Biology, UNAM).

## Conflict of Interest

The authors declare that the research was conducted in the absence of any commercial or financial relationships that could be construed as a potential conflict of interest.
