# Supplementary material for: Fas/FasL Signaling Regulates CD8 Expression During Exposure to Self-Antigens
Source: Front Immunol. 2021 Mar 24;12:635862. doi: 10.3389/fimmu.2021.635862 (PMC8024570; doi:10.3389/fimmu.2021.635862)
Supplement: Supplementary file 1 [file DataSheet_1.pdf]

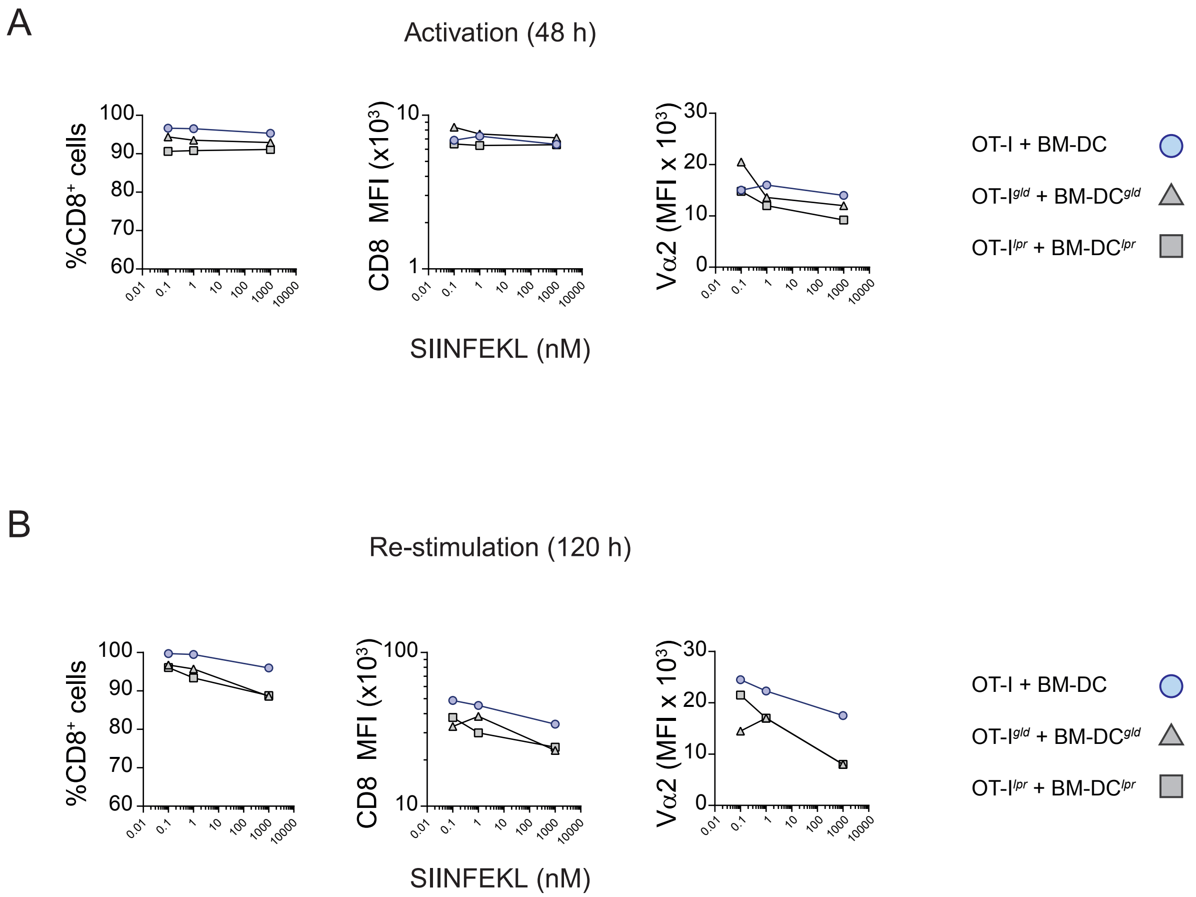

Supplemental Figure 1. Strength of TCR signaling during activation and re-stimulation of CD8 T cells. Wild-type, Fas- (*lpr*), or FasL-deficient (*gld*) CD8$^+$ OT-I cells were activated in the presence of WT, *lpr*, or *gld* BM-DCs loaded with varying concentrations of the ovalbumin-derived peptide SIINFEKL (0.1-1000 nM). After 48 hours (activation), CD8 expression levels were quantified by flow cytometry in an aliquot of the cells. The rest were placed in a new plate with fresh BM-DCs (in the presence of SIINFEKL). CD8 expression was reevaluated after 72 hours (120 h re-stimulation). Percentage of CD8$^+$ cells (left) or CD8 expression per cell (mean fluorescence intensity; MFI; middle), or TCR-Vα2 expression per cell (right), on OT-I cells is shown.

Supplemental Table 1. Flow cytometry antibodies

Target	Fluorochrome	Clone	Catalog number and company
**CD8a**	PE	53-6.7	50-0081, Tonbo Biosciences
	PerCP-Cyanine5.5	53-6.7	65-0081, Tonbo Biosciences
	FITC	53-6.7	35-0081, Tonbo Biosciences
	redFluor 710	53-6.7	80-0081, Tonbo Biosciences
**TCR Vα2**	FITC	B20.1	127805, BioLegend
	Pacific Blue	B20.1	127816, BioLegend
	PE	B20.1	127808, BioLegend
**TCR Vβ5**	PE/Cyanine7	MR9-4	139508, BioLegend
**CD44**	violetFluor 450	IM7	75-0441, Tonbo Biosciences
	FITC	IM7	35-0441, Tonbo Biosciences
**CD45.1**	APC-Cyanine 7	A20	25-0453, Tonbo Biosciences
	FITC	A20	35-0453, Tonbo Biosciences
	Brilliant Violet 605	A20	110738, BioLegend
**CD45.2**	APC-Cyanine 7	104	25-0454, Tonbo Biosciences
	APC	N418	20-0114, Tonbo Biosciences
	violetFluor 450	104	75-0454, Tonbo Biosciences
**CD11c**	APC	N418	17-0114-81, eBiosciences
**I-A/I-E**	Pacific Blue	M5/114.15.2	107620, BioLegend
**Fas**	APC	15A7	17-0951-82, Invitrogen
**FasL**	PE	MFL3	106606, BioLegend
